# Pediatric Pilonidal Disease Surgical Approach (PPiDSA) Study

**DOI:** 10.3390/children13020254

**Published:** 2026-02-12

**Authors:** Marta Rodríguez Ruiz, Isabella Garavis Montagut, Inmaculada Ruiz Jiménez, Noela Carrera, Pablo Aguado Roncero, Ennio Fuentes, Ricardo Díez, Carlos Delgado-Miguel

**Affiliations:** 1 Pediatric Surgery Department, Fundación Jiménez Díaz University Hospital, 28040 Madrid, Spainrdiez@quironsalud.es (R.D.); 2 Faculty of Medicine, El Bosque University, Bogotá 28040, Colombia; 3 Pediatric Surgery Department, Villalba University Hospital, 28400 Villalba, Spain; 4 Pediatric Surgery Department, Infanta Elena University Hospital, 28342 Valdemoro, Spain; 5 Pediatric Surgery Department, Rey Juan Carlos University Hospital, 28933 Móstoles, Spain; 6 Institute for Health Research IdiPAZ, La Paz University Hospital, 28046 Madrid, Spain

**Keywords:** pilonidal sinus, pediatrics, endoscopy, minimally invasive surgical procedures

## Abstract

**Background:** Wide surgical excision is considered the traditional treatment for pilonidal sinus disease but is associated with prolonged wound healing and delayed recovery. This study aims to compare the outcomes of radical surgery versus a minimally invasive alternative in pediatric patients. **Methods:** A multicenter randomized comparative study was conducted on patients undergoing surgery for pilonidal sinus between January 2023 and December 2024 in four pediatric institutions. Patients were divided into two groups according to the surgical approach: Group 1 (en bloc excision) and Group 2 (endoscopic treatment). Demographic and clinical variables, postoperative complications, and recurrence rates were analyzed. **Results:** A total of 102 patients were included in this study (Group 1: n = 54; Group 2: n = 48). Baseline demographic and clinical characteristics were similar between groups. Postoperative complications occurred more frequently in Group 1 compared with Group 2 (53.7% vs. 20.8%; *p* < 0.001). Moreover, Group 1 presented a higher proportion of emergency department visits (44.4% vs. 8.36%; *p* < 0.001) and a longer median time to complete healing (100 vs. 45 days; *p* < 0.001). After a median follow-up of 19 months (interquartile range, 10–24 months), no significant differences were observed between groups in recurrence rates (14.8% vs. 16.7%; *p* = 0.797) or in median time from treatment to recurrence (198 vs. 184 days; *p* = 0.672). **Conclusions:** Endoscopic treatment of pilonidal sinus in pediatric patients is associated with fewer postoperative complications and faster recovery compared to radical surgery, with no observed differences in recurrence rates. Longer follow-up is needed to confirm these findings.

## 1. Introduction

Since its initial description over a decade ago in adult patients, endoscopic pilonidal sinus Treatment (EPSiT) has steadily gained acceptance due to its less invasive nature and reduced associated morbidity compared to traditional open surgery [1]. These benefits encouraged the adoption of the technique in pediatric patients (PEPSiT) over the past few years [2]. Current evidence supports PEPSiT as a minimally invasive and well-tolerated approach for children. It is associated with shorter operative times less reported wound complications, faster resumption to daily activities, and higher levels of patient satisfaction compared to traditional open excisional methods such as en-bloc excision with healing by secondary intention and en-bloc excision with primary closure [3].

However, recurrence rates remain a concern when compared with excisional methods that have demonstrated better long-term disease control [4]. While these techniques may help reduce recurrence rates, they are associated with longer operative times and increased early morbidity, primarily due to prolonged wound healing and recovery times [3,5]. To date, most studies on PEPSiT in pediatric populations consist of single- or multicenter observational case series with heterogeneous lengths of follow-up [6,7]. However, there is a notable lack of randomized studies comparing this technique to en-bloc excision [8,9]. The aim of this study is to evaluate the short- and long-term outcomes of endoscopic treatment and compare them with en-bloc excision without primary closure (healing by secondary intention) through a randomized study design, in order to minimize potential patient selection bias.

## 2. Methods

### 2.1. Study Design

A multicenter study was conducted involving patients under 18 years of age with pilonidal sinus disease, who were treated between January 2023 and December 2024 in four pediatric centers (Fundación Jiménez Díaz University Hospital, Rey Juan Carlos University Hospital, Villalba University Hospital, Infanta Elena University Hospital). Patients were assigned between two groups based on the surgical treatment received as follows: the EWE group (en-bloc wide excision without primary closure) and the PEPSiT group (endoscopic treatment). Patient distribution was performed using a randomization system generated by Microsoft Excel 2020 (Redmond, WA, USA) with the =RANDBETWEEN(0,1) function. Assignment was not concealed, in order to inform the patients and families about postoperative care. Patients who declined participation or had incomplete follow-up after the intervention were excluded.

The primary outcome was to assess and to compare recurrence rates of pilonidal sinus disease between the two groups. Secondary outcomes included: postoperative complications (wound infection, discharge, bleeding, or need for emergency department visits), and impact on daily activities (measured by the number of days until return to routine activities and time to medical discharge).

### 2.2. Data Analysis

Demographic, clinical, and postoperative data were prospectively collected during follow-up visits. Demographic variables included age at the time of surgery, height, weight, body mass index (BMI), and BMI Z-score, calculated according to reference values from the Spanish Growth Studies published by Carrascosa et al. [10]. Clinical variables analyzed included: the presence of cutaneous abscess at the time of surgery, number of midline pits, and presence of a gluteal fistula. Postoperative outcomes encompassed both short-term complications occurring within the first month after surgery and long-term outcomes including disease recurrence at the end of the study period. Postoperative complications comprised wound infection, postoperative wound discharge, bleeding, and emergency department visits. Wound infection was defined by localized erythema and/or edema associated to purulent discharge; drainage of serous fluid was not considered infectious. Recurrence was defined as the reappearance of pilonidal disease symptoms after a symptom-free interval following complete wound healing. In cases of recurrence, surgical reintervention or a conservative approach were offered, with the final decision based on clinical status and family and patient preference.

Number of days until return to normal activities, time to medical discharge (measured in number of days), recurrence of pilonidal disease, and need for reoperation were collected. Each patient underwent weekly outpatient evaluations by the operating surgeon starting two weeks after surgery, and subsequently at 1, 3, 6, and 12 months postoperatively. A successful outcome was defined as complete wound healing and the absence of any patient-reported symptoms. Complete wound healing was defined as full epithelialization with absence of drainage and no need for dressings at two consecutive assessments. Medical discharge was defined as the time point at which no further scheduled wound care or follow-up visits were required, unless new symptoms suggestive of recurrence developed. Follow-up was conitnued until complete wound healing or the need for additional intervention. Long-term follow-up and recurrence of disease were monitored through outpatient clinic visits. Postoperative complications were additionally graded according to the Clavien–Dindo classification. Complication grading was based on the treatment required (e.g., bedside care only, antibiotics, procedural intervention, readmission, or reoperation), enabling standardized comparison across groups. An updated assessment of the patients clinical status was conducted via telephone in June 30, 2025, to identify any late recurrences not detected during routine clinical visits.

### 2.3. Surgical Aspects

All patients were admitted on the day of surgery. Prophylactic antibiotics were not routinely administered according to institutional protocols and antimicrobial stewardship policies; antibiotics were reserved for clinically suspected infection. Neither the operative technique nor the anesthetic protocol was modified during the course of the study. All procedures were performed by a predefined team of six pediatric surgeons across the participating centers. Surgeons were assigned to a single approach (EWE or PEPSiT) based on prior experience and specific training in that technique, aiming to minimize learning-curve effects and standardize operative performance. Three surgeons performed all procedures in the EWE group, whereas a different set of three surgeons operated exclusively on patients in the PEPSiT group.

### 2.4. Surgical Procedures

Patients in the EWE group underwent surgery in a prone position under general anesthesia with orotracheal intubation. An en-bloc excision of the lesion was performed through an elliptical midline intergluteal incision, following the injection of methylene blue to delineate the fistulous tracts. In cases with secondary cavities or lateral extensions, the resection was extended to include all affected tissue until healthy margins were achieved. In all patients, dissection was carried down to the sacrococcygeal fascia, and the specimen was sent for histopathological analysis. Hemostasis was then achieved, and an occlusive dressing with nitrofurantoin-impregnated gauze was applied, but no primary wound closure was performed. In contrast, patients in the PEPSiT group were also positioned prone but operated on under sedation and local anesthesia. After antiseptic preparation of the area, the fistulous tract was gently dilated using a curved clamp to allow the introduction of a pediatric cystoscope, as a dedicated fistuloscope was not available. The affected cavities were identified and hair was removed under direct endoscopic visualization. Mechanical abrasion of the cavity walls was then performed using a bronchoscopy brush, followed by monopolar electro-fulguration of the fistulous tract. Finally, a gauze tip impregnated with nitrofurantoin was placed inside the cavity. Postoperative wound care was identical in both groups and consisted of daily cleansing with aqueous chlorhexidine, followed by topical antibiotic application (nitrofurantoin) and occlusive gauze dressing. Families also received standardized written instructions on hair control and cleft hygiene after healing, including regular hair removal of the intergluteal region and avoidance of prolonged moisture/friction; however, adherence to hair-control measures was not formally quantified. Follow-up in the outpatient clinic was also conducted similarly for both groups, as previously described.

### 2.5. Ethical Aspects

The study protocol was adherent to Declaration of Helsinki guidelines, as updated in successive world assemblies (most recently in Fortaleza, Brazil, October 2013), and complied with Good Clinical Practice standards. Ethical approval was obtained from the hospital’s institutional review board (IRB number PI-272-24-FJD). Written informed consent was obtained from the patients’ parents or legal guardians in every instance. All data collected during the study were treated as confidential and handled in full compliance with current data protection regulations, including the European Parliament and Council Regulation (EU) 2016/679 of 27 April 2016 (GDPR), and the Spanish Organic Law 03/2018 on data protection.

### 2.6. Statistical Analysis

Data collection was performed using Microsoft Excel 2010 (Redmond, WA, USA), and statistical analyses were conducted with IBM’s Statistical Package for the Social Sciences (SPSS), version 25.0 (Armonk, NY, USA). Categorical variables were presented as frequencies and percentages, and comparisons were made using the Chi-square test or Fisher’s exact test when appropriate. The distribution normality of variables was evaluated through the Kolmogorov-Smirnov and Shapiro-Wilk tests. For parametric data exhibiting a normal distribution, the Student’s *t*-test was applied (results expressed as mean ± standard deviation). When normality was not found, non-parametric Mann-Whitney U tests were used, with results reported as median and interquartile range (IQR). Relative risk (RR) estimates with 95% confidence intervals were also calculated. Recurrence-free survival and time to reintervention were estimated using Kaplan-Meier survival curves and compared via the log-rank test. All statistical tests were two-sided, and a *p*-value less than 0.05 was considered statistically significant.

A post hoc power analysis was performed to evaluate whether the sample size was adequate to detect a clinically meaningful difference in recurrence rates between open and endoscopic surgical techniques. The analysis was conducted using a two-proportion test implemented via the power.prop.test function in R software (R Core Team, Vienna, Austria), assuming recurrence rates of 5% for the open surgery group and 20% for the endoscopic surgery group, with a two-sided alpha level of 0.05.

## 3. Results

A total of 102 patients were included (63 males and 39 females), with a mean age of 15 ± 1.64 years and a mean BMI of 25.6 ± 5. Of these, 54 patients underwent en-bloc wide excision without primary closure (EWE group), and 48 were treated using the endoscopic technique (PEPSiT group). The post hoc power analysis confirmed that the sample sizes of 54 and 48 patients in each group provided approximately 82% power to detect such a difference at a significance level of 0.05 (two-sided). This supports the adequacy of the sample size for identifying clinically relevant differences in recurrence rates between the surgical methods. No significant demographic differences were found between the two groups in terms of age, sex distribution, weight, height, or BMI. At the time of surgery, 26.5% of patients presented with a cutaneous abscess, with a median number of midline pits of 2 (IQR: 1–3), and 54.9% exhibited a laterally located gluteal fistula. Comparative analysis of the clinical characteristics between the two groups, showed no significant statistical differences. Details are summarized in Table 1. Regarding postoperative outcomes, complications were observed in 29 patients (53.7%) in the EWE group, representing a significantly higher rate compared to PEPSiT group, in which 10 patients (20.8%) presented some type of complications [RR 2.58 (1.41–4.72), *p* < 0.001]. Regarding complications according to Clavien-Dindo (CD) classification, postoperative wound discharge (CD grade 1) was reported in 9 EWE patients (16.7%) compared to 7 PEPSiT cases (14.6%) without statistically significant variation between groups. Wound infection (CD grade 2) occurred in 14 EWE patients (25.9%) and in 6 PEPSiT patients (12.5%) without significant difference. Bleeding (CD grade 2) occurred in 6 EWE patients (12.9%) while in 0 PEPSiT patients (0%) without significant difference; and the proportion of patients requiring emergency department visits was significantly higher in the EWE group (44.4%) compared to the PEPSiT group (8.3%) [RR = 5.33; 95% CI: 1.99–14.26; *p* < 0.001]. Recovery metrics also favored the PEPSiT group: the median number of days without daily activity and days until medical discharge were significantly greater in the EWE group (71 and 100 days, respectively) than in the PEPSiT group (18 and 45 days, respectively; *p* < 0.001 for both comparisons). Postoperative outcomes for both groups are summarized in Table 2.

**Figure 1:**
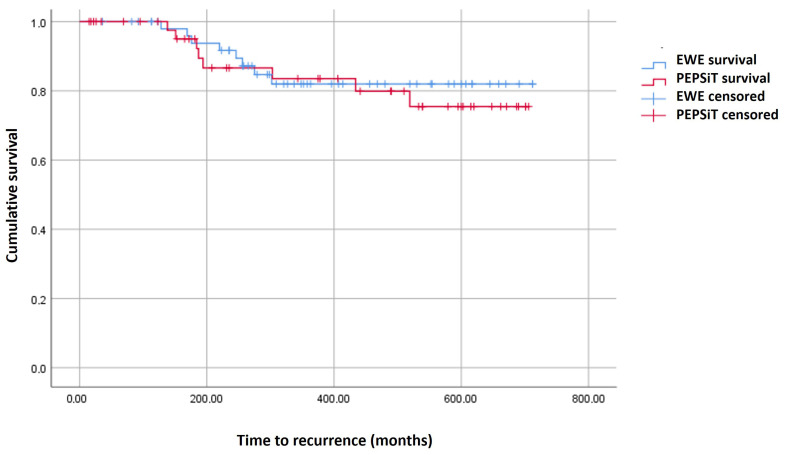
Kaplan-Meier curve comparing en-bloc wide excision without primary closure (EWE group), and endoscopic technique (PEPSiT) group.

Finally, during long-term follow-up, a total of 16 recurrences (15.7%) were observed, with a median time of 19 months (IQR 10–24). Recurrences occurred in 8 patients from each group (14.8% in the EWE group and 16.7% in the PEPSiT group, *p* = 0.797). No significant differences were found in the median number of days until recurrence (198 days in the EWE group vs. 184 days in the PEPSiT group). Time to recurrence was analyzed using a Kaplan–Meier survival curve (Figure 1), which showed no statistically significant difference between the two groups (log-rank test = 0.163). There were 11 reinterventions in patients with recurrences, 5 in the EWE group (9.3%) and 6 in the PEPSiT group (12.5%), without this difference being statistically significant. Table 3 summarizes the long-term outcomes in terms of recurrence and the need for reintervention.

## 4. Discussion

PEPSiT technique has emerged as a minimally invasive alternative to conventional open excision surgery in recent years [5]. Unlike traditional open surgery, PEPSiT does not involve wide excision of the affected tissue; instead, it focuses on endoscopic identification and destruction of the sinus tracts with minimal disruption of surrounding structures. This approach has been associated with significant advantages in terms of postoperative recovery, including reduced pain, shorter time away from daily activities, faster wound healing, and improved cosmetic outcomes [1,11]. However, concerns regarding the long-term efficacy of PEPSiT remain. Since this technique avoids complete excision of the pilonidal area, there is a theoretical risk of leaving behind microscopic disease or unidentified tracts, which could increase the likelihood of recurrence and the need for reintervention [11,12]. In contrast, open excision surgery aims to completely remove all affected tissue, potentially reducing the recurrence rate. This benefit, however, is offset by longer wound healing times, more intense postoperative care, and greater disruption of normal activities.

Most published studies on the effectiveness of PEPSiT are descriptive case series with varying sample sizes and follow-up durations. The first series in pediatric patients was published in 2018 by Esposito et al., reporting 15 patients with no recurrences observed at 6 months of follow-up. Shortly after, they published a multicenter study involving 43 patients treated with PEPSiT, reporting a 9% complication rate and a 12% recurrence rate, with a median follow-up of 4 months [13]. Subsequently in 2020, they implemented a protocol to perform laser epilation both pre- and postoperatively in all patients, reporting a recurrence rate of 1.6% among a total of 59 patients [14]. One of the main limitations of these studies is the absence of a control group, which hinders the generalizability of the results. More recently same researchers, presented a multicenter study of 294 patients with a recurrence rate of 4.8%, although their cohort included both primary procedures and 36 reinterventions, without stratifying the outcomes by group [6]. This lack of stratification may lead to potential misperceptions of the results. In contrast, our study included only patients undergoing their first surgical intervention, explicitly excluding recurrent cases, in order to minimize confounding factors and ensure a clearer and more accurate evaluation of outcomes specific to primary PEPSiT procedures.

There are few comparative studies conducted in pediatric patients, nevertheless, the available evidence shows conflicting results regarding the effectiveness of the endoscopic technique. Lee et al. retrospectively compared 45 patients treated with PEPSiT versus 18 patients who underwent excision with off-midline flap reconstruction using the Bascom cleft lift flap, a Limberg flap modification [12]. They observed that patients in the reconstruction cohort had a higher rate of wound complications (22.2% vs. 0%; *p* = 0.001), but significantly lower rates of disease recurrence (5.6% vs. 33.3%; *p* = 0.022) and reoperation (5.6% vs. 31.1%; *p* = 0.031). In contrast, the results reported by Pérez-Bertólez et al. showed a 5% recurrence rate in 100 patients treated with PEPSiT, compared to 29% in 21 patients treated with excision and primary closure, and 18% in 28 patients treated with excision without closure [8]. However, the study presents some limitations due to the lack of randomization, as the surgical technique used in each case was selected at the discretion of the individual surgeon. Additionally, one-third of the patients in the PEPSiT group underwent laser hair removal—an intervention known to reduce the risk of recurrence—compared to only 10% and 18% in the two open surgery groups, this might have created a potential selection bias that may have influenced the outcomes.

In this context, a randomized comparative study such as the present one is essential to accurately assess the true effectiveness and safety of PEPSiT compared to conventional open excision. To date, most available comparative studies in the literature are retrospective or non-randomized, which inherently introduces a high risk of selection bias. In such studies, physicians might select patients with less severe or more favorable disease profiles for minimally invasive approaches like PEPSiT, while reserving more complex or extensive cases for open surgery. This may result in an overestimation of the benefits of PEPSiT and an inadequate portrayal of its limitations, particularly in terms of recurrence and reintervention rates. Randomization ensured balanced groups and minimized selection bias, allowing a more reliable comparison of postoperative outcomes, recurrence, and reintervention rates. The effectiveness of this approach is reflected in the homogeneity observed between the two study groups. By minimizing confounding variables, randomization allows a more rigorous assessment of each technique’s outcomes, including postoperative recovery, complications, recurrence and reintervention rates. These findings therefore might provide valuable information regarding the effectiveness and limitations of each surgical strategy, contributing valuable data to inform future clinical decision-making and guideline development. Also, its multicenter design contributes to greater external validity, and the application of appropriate statistical analysis, which allowed the identification of significant differences and ensured a robust study. Anesthetic management differed between groups, with open surgery performed under general anesthesia and PEPSiT under sedation and local anesthesia. Although this reflects routine clinical practice and is inherent to each technique, it represents a potential confounding factor and may limit the ability to isolate the effect of the surgical approach itself when interpreting the results.

Regarding postoperative complications, our study demonstrated a significant reduction in the PEPSiT group (20.8%) compared to open surgery (53.7%). These rates are below thosereported by Pérez-Bertólez et al., who observed a 24% complication rate in the PEPSiT group and 72% in patients who underwent excision without primary closure [8]. Esposito et al. reported an even lower rate of 2%, although they only considered complications of grade II or higher according to the Clavien-Dindo classification [6]. An important aspect to consider is that postoperative wound care was identical for both groups included daily cleansing with chlorhexidine, topical antibiotic application (nitrofurantoin), and occlusive gauze dressing. This standardized postoperative management likely minimized confounding bias in postoperative infectious outcomes in our cohort.

In terms of postoperative recovery, our study showed that PEPSiT allows faster convalescence, reflected by fewer days of restricted daily activities (15 days vs. 88 days) and a shorter time to medical discharge (45 days vs. 99 days). These outcomes also surpass those reported by Pérez-Bertólez et al. [8] (22 days vs. 74 days, respectively), and by Esposito et al. [6], who analyzed only PEPSiT procedures and reported a mean of 23.4 days for patients to return to daily activities. No significant differences were found in terms of recurrence rates or the need for reoperation between endoscopic surgery and open surgery without primary closure. This type of randomized comparison has not been conducted in any previously published studies, representing a profitable contribution. These findings challenge the theory that endoscopic surgery carries a higher risk of recurrence than open surgery, as both treatments were applied to a homogeneous sample in a randomized fashion, without prior laser hair removal or other preoperative treatments. The recurrence rate observed in the PEPSiT group is comparable to that reported by other authors [15,16], although some descriptive series have shown lower recurrence rates than those found in our study, these generally involved shorter follow-up periods. For example, Sequeira et al. reported a 9.5% recurrence rate with a median follow-up of 11 months [9], while Dotlacil et al. observed an 11.7% rate with a median follow-up of 10.2 months [7]. Erculiani et al. noted that recurrence rates increased over time, with 8% at 6 months and 15% at 19 months postoperatively. Our findings were consistent with those reported by Erculiani et al. obtained with a similar follow-up period, making remarkable the adequate long-term postoperative follow-up to reliably assess the effectiveness of endoscopic surgical techniques.

Some authors have reported that the length of hospital stay was significantly lower in the PEPSiT group, as the procedure was performed as ambulatory surgery, while open surgery required hospitalization in some cases [8]. Most descriptive series in pediatric patients report a hospital stay length of approximately 24 h following PEPSiT [7,9,13]. In our study, all patients underwent surgery in an ambulatory setting and were discharged the same day without need for hospitalization, regardless of the technique used, and postoperative care was carried out following the same standardized protocol previously described. Therefore, we did not observe any differences in this aspect between the groups.

We found certain limitations in this study that should be acknowledged. First, for outcomes such as recurrence and reintervention, the statistical power could be improved with a larger sample size or a longer follow-up period. Additionally, all participating hospitals are located in the central region of Spain, this geographical restriction may limit the extrapolation of findings in other populations, and external validation would be necessary before broader application. The study would have benefited from the inclusion of patient-reported pain scales (such as visual analog scale and analgesic requirements) and data on adherence to postoperative care. Future studies will benefit from incorporating shared surgical teams with standardized anesthesia protocols. Moreover, the absence of standardized or documented hair control protocols were not objectively quantified, limiting interpretation of recurrence. These factors would provide additional insights into the overall benefits of each surgical technique and their impact on patient quality of life. Finally, evaluating the cost-effectiveness of both approaches, as well as assessing patient and family satisfaction using standardized quality-of-life questionnaires, would offer a more comprehensive understanding of the outcomes and value of each intervention.

## 5. Conclusions

The results of our study suggest that endoscopic treatment of pediatric pilonidal sinus disease may reduce early postoperative burden compared with en-bloc wide excision without primary closure, including fewer postoperative complications, fewer emergency department visits, and faster functional recovery with earlier medical discharge. These findings highlight the potential of the endoscopic technique to improve patient outcomes and reduce the burden of postoperative care. Importantly, recurrence and reintervention rates were similar between techniques within the available follow-up; therefore, superiority in long-term disease control cannot be concluded from these data. Given potential performance bias related to surgeon separation and different anesthesia protocols, as well as the lack of patient-reported outcomes (pain, satisfaction, quality of life), further multicenter studies with standardized endpoints and longer follow-up are required to confirm durability and overall patient benefit of this minimally invasive approach.

## Figures and Tables

**Table 1:** Demographic and clinical features of patients in both groups.

	Total (n = 102)	EWE Group (n = 54)	PEPSiT Group (n = 48)	*p*-Value
**Age (years), mean (SD)**	15.0 (1.64)	14.7 (1.8)	15.3 (1.4)	0.228
**Gender, n (%)**				
**Male**	63 (61.8)	34 (63.0)	31 (64.6)	0.865
**Female**	39 (38.2)	20 (37.0)	17 (35.4)	
**BMI (kg/m^2^), mean (SD)**	72.3 (15.9)	71.4 (15.6)	73.7 (15.7)	0.569
**Cutaneous abscess, n (%)**	27 (26.5)	19 (35.2)	10 (20.8)	0.109
**Midline pits, median (IQR)**	2 (1–3)	1 (1–3)	2 (1–3)	0.264
**Gluteal fistula, n (%)**	56 (54.9)	37 (68.5)	24 (50.0)	0.057

EWE, en-bloc wide excision; PEPSiT, Pediatric Endoscopic Pilonidal Sinus Treatment; SD, standar deviation; BMI, Body Mass Index; IQR, interquertile range.

**Table 2:** Comparison of postoperative outcomes between both groups.

	Total (n = 102)	EWE Group (n = 54)	PEPSiT Group (n = 48)	RR (95% CI)	*p*-Value
**Total complications; n (%)**	39 (38.2)	29 (53.7)	10 (20.8)	2.58 (1.41–4.72)	<0.001
**Infection; n (%)**	20 (19.6)	14 (25.9)	6 (12.5)	2.07 (0.87–4.97)	0.133
**Bleeding; n (%)**	6 (5.9)	6 (12.9)	0	-	0.013
**Wound discharge; n (%)**	16 (15.7)	9 (16.7)	7 (14.6)	1.14 (0.45–2.89)	0.787
**Required ED visit; n (%)**	28 (22.5)	24 (44.4)	4 (8.3)	5.33 (1.99–14.26)	<0.001
**Days without daily activity (IQR)**	58 (15–98)	71 (57–129)	18 (15–25)	-	<0.001
**Days to medical discharge (IQR)**	79 (44–157)	100 (71–175)	45 (34–85)	-	<0.001

EWE, en-bloc wide excision; PEPSiT, Pediatric Endoscopic Pilonidal Sinus Treatment; RR, relative risk; CI, confidence Interval; ED, Emergency Department, IQR, interquertile range.

**Table 3:** Comparison of recurrence and reoperation rates between both groups.

	Total (n = 102)	EWE Group (n = 54)	PEPSiT Group (n = 48)	RR (95% CI)	*p*-Value
**Recurrences, n (%)**	16 (15.7%)	8 (14.8%)	8 (16.7%)	0.880 (0.36–2.18)	0.797
**Days to recurrence, median (IQR)**	185 (117–270)	198 (105–260)	184 (36–303)	–	0.672
**Reoperations, n (%)**	11 (10.7%)	5 (9.3%)	6 (12.5%)	0.741 (0.203–2.509)	0.598

EWE, en-bloc wide excision; PEPSiT, Pediatric Endoscopic Pilonidal Sinus Treatment; RR, relative risk; CI, confidence Interval; IQR, interquertile range.

## Data Availability

The data presented in this study are available on request from the corresponding author. The data are not publicly available due to restrictions privacy.
